# Blue Rubber Bleb Nevus Syndrome: Case-Driven Insights Into a Rare Cause of Refractory Gastrointestinal Bleeding

**DOI:** 10.7759/cureus.104842

**Published:** 2026-03-07

**Authors:** Edwin Ornelas Escobedo, Laura V Cupil Escobedo, Santiago Philibert-Rosas, Ceriolith Tenorio Flores, Ariosto H Hernandez Lara, Luis E Zamora Nava

**Affiliations:** 1 Gastroenterology, Mexico's General Hospital "Dr. Eduardo Liceaga", Mexico City, MEX; 2 Medicine, University of Wisconsin School of Medicine and Public Health, Madison, USA; 3 Gastrointestinal Endosocpy, Mexico's General Hospital "Dr. Eduardo Liceaga", Mexico City, MEX; 4 Surgery, Hospital Angeles Lomas, Mexico City, MEX; 5 Gastroenterology, Salvador Zubirán National Institute of Health Sciences and Nutrition, Mexico City, MEX

**Keywords:** blue rubber bleb nevus syndrome, hot snare polypectomy, refractory gastrointestinal bleeding, therapeutic endoscopy, video capsule endoscopy

## Abstract

Blue rubber bleb nevus syndrome (BRBNS) is a rare vascular disorder causing chronic iron-deficiency anemia from gastrointestinal (GI) bleeding. We described the case of a 17-year-old Latin American patient with refractory anemia, cutaneous papules, and blue nevi. Endoscopic evaluations identified multiple bleeding lesions through the GI tract. Hot snare resection and clip placement achieved significant clinical improvement and stabilized hemoglobin levels. This case underscores the critical role of video capsule endoscopy and highlights the need for heightened awareness and diagnostic expertise in managing BRBNS.

## Introduction

Blue rubber bleb nevus syndrome (BRBNS), or bean syndrome, is a congenital disorder characterized by multiple venous malformations, primarily affecting the skin and gastrointestinal (GI) tract. It is rare, with approximately 200 cases reported in the literature. These lesions are slow-flow venous malformations, historically termed “cavernous hemangiomas,” which led earlier literature to describe BRBNS lesions as “hemangiomas” despite their venous malformation biology [1]. Although the precise mechanism of lesion formation in BRBNS remains incompletely defined, most cases appear sporadic; familial cases have been reported, and genetic susceptibility has been proposed, including a locus on chromosome 9p in dominantly inherited venous malformations. Activating mutations in the endothelial receptor tyrosine kinase TIE2 (TEK) at this locus have been shown to cause venous malformations, though TIE2/TEK mutations have not been directly linked to BRBNS to date [2].

However, they can also involve other organ systems such as the liver, spleen, heart, eye, and central nervous system (CNS) [1,3,4]. These venous malformations often present as soft, compressible, dark blue papules on the skin. Still, their most significant clinical manifestation occurs in the GI tract, where they can lead to occult or massive GI bleeding, resulting in severe iron-deficiency anemia [5]. In patients with refractory iron-deficiency anemia or obscure GI bleeding, angiodysplasia and other vascular lesions should be considered, and small-bowel evaluation may be required when esophagogastroduodenoscopy (EGD) and colonoscopy are non-diagnostic. 

The management of BRBNS is complex and requires a multidisciplinary approach involving hematology, dermatology, gastroenterology, and other specialties. Treatment is mainly supportive, focusing on the management of complications such as volvulus, intussusception, infarction, and recurrent GI bleeding. Endoscopic intervention may be necessary for refractory cases of GI hemorrhage, particularly when traditional medical management is insufficient to control the anemia associated with recurrent blood loss [2]. Here, we report a GI-predominant BRBNS presentation with refractory anemia requiring small-bowel evaluation and endoscopic therapy. 

## Case presentation

**Figure 1:**
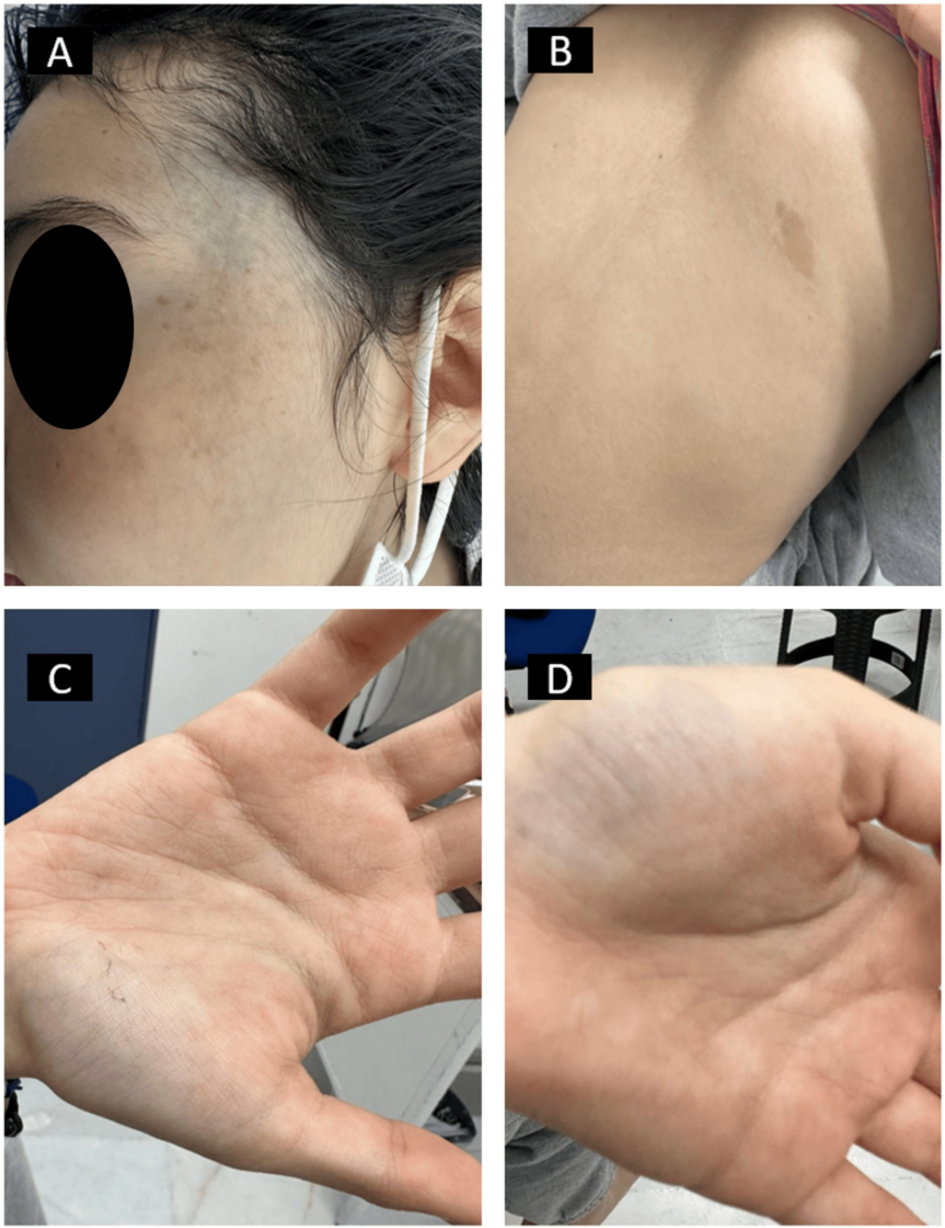
(A) Close-up of a bluish papule on the patient's head. (B) Back view showing a raised lesion. (C) Right hand with small, blue-tinted nevi. (D) Left hand with a similar small, blue nevi lesion.

**Figure 2:**
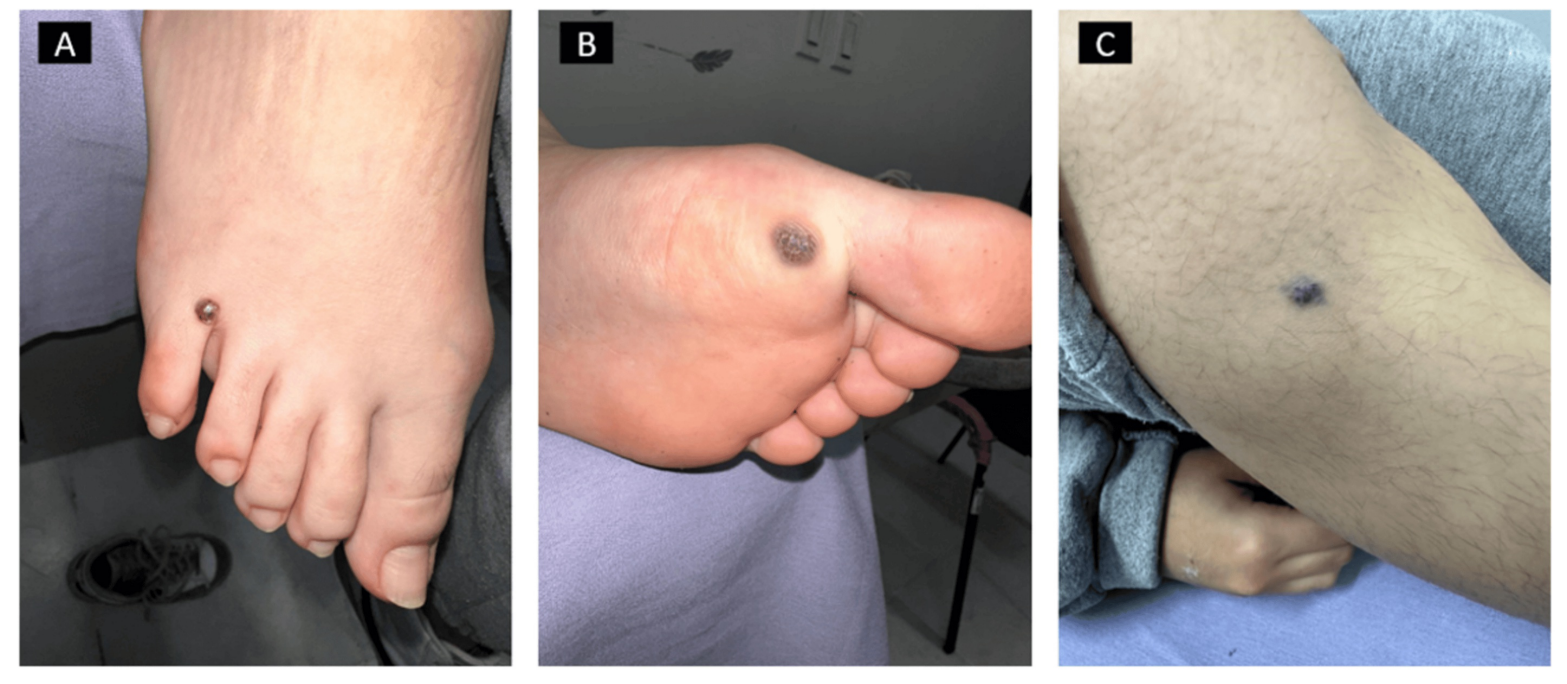
(A) Raised, dark nevus on the top of the right foot. (B) Large, bluish lesion on the sole of the left foot. (C) Small, dark lesion on the tibial face of the right lower extremity.

A 17-year-old female patient presented with a five-year history of persistent anemia, unresponsive to standard iron supplementation. On physical examination, distinctive cutaneous findings were noted (Figure 1), including multiple papules located bilaterally in the frontal region and prominent blue nevi on both palms and soles (Figure 2). These dermatological manifestations prompted further investigation.

Initial diagnosis workup included a colonoscopy, which identified two polypoid lesions in the sigmoid and ascending colon. These lesions were successfully excised during the procedure. A concurrent EGD was performed, which did not reveal any significant abnormalities in the upper GI tract.

The patient underwent treatment with IV iron therapy, resulting in partial improvement of her anemia. However, her condition remained refractory, needing periodic red blood cell transfusions despite the absence of overt GI bleeding. Subsequent fecal occult blood testing was positive, raising suspicion for a potential GI blood loss. This prompted a stepwise GI evaluation with bidirectional endoscopy (EGD and colonoscopy), followed by small-bowel assessment when initial studies were non-diagnostic. 

**Figure 3:**
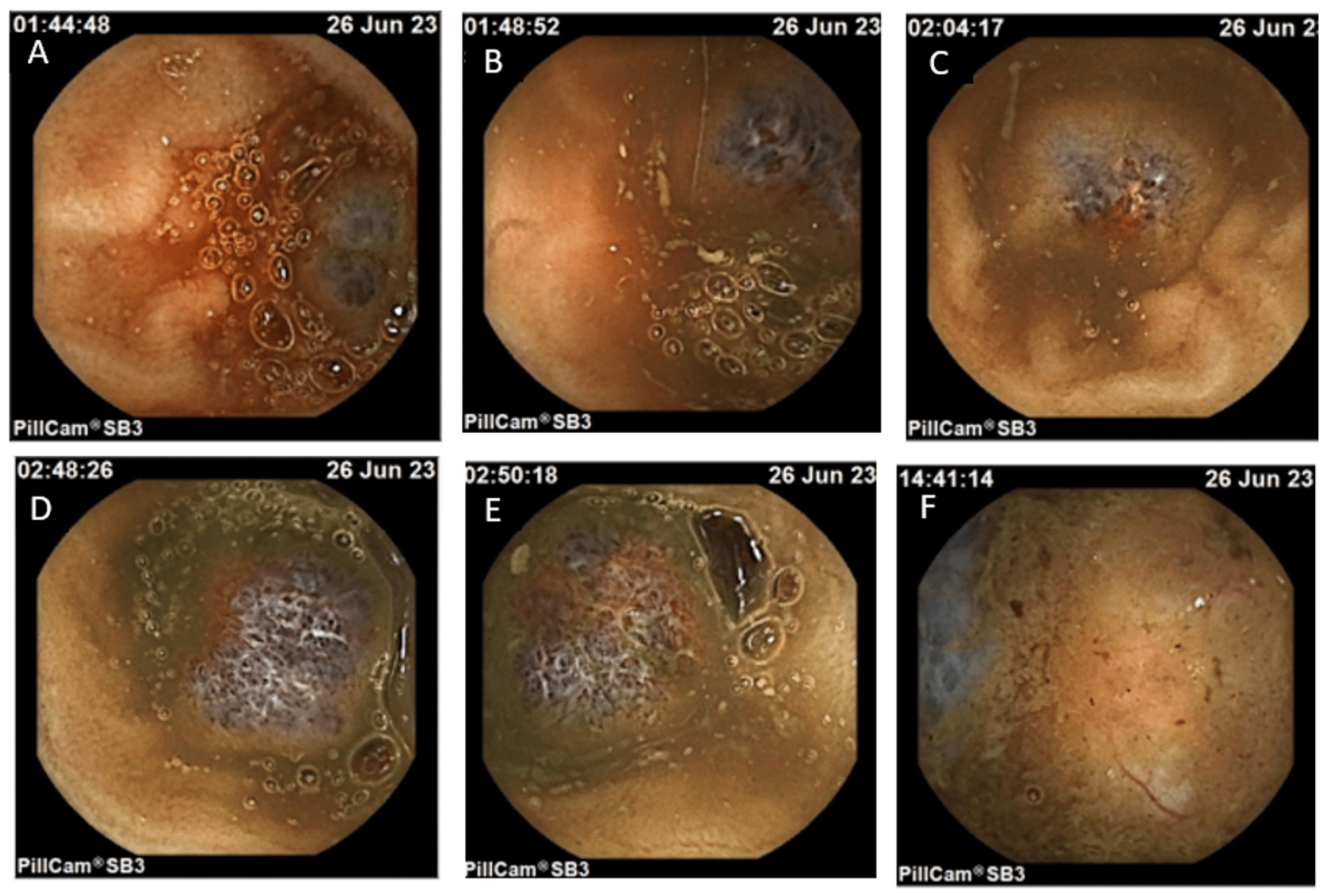
(A) Vascular, nodular, polypoid neoformation measuring approximately 15-20 mm, showing active hemorrhage. (B) Vascular, nodular lesions with a polypoid appearance, though no active bleeding is evident here. (C) Vascular neoformation with slight active hemorrhage and a nodular, polypoid aspect. (D, E) Vascular nodular formations (15-20 mm) filling most of the lumen. (F) Vascular nodular formation without obvious signs of bleeding in these frames.

Given concern for a small-bowel bleeding source after a non-diagnostic upper endoscopy and colonoscopy, a video capsule endoscopy was conducted, which revealed six vascular lesions with a polypoid appearance distributed throughout all segments of the small intestine. Notably, active bleeding was observed from two lesions located in the distal ileum (Figure 3). These findings required therapeutic intervention. Accordingly, device-assisted enteroscopy was pursued to localize and treat the bleeding lesions endoscopically.

**Figure 4:**
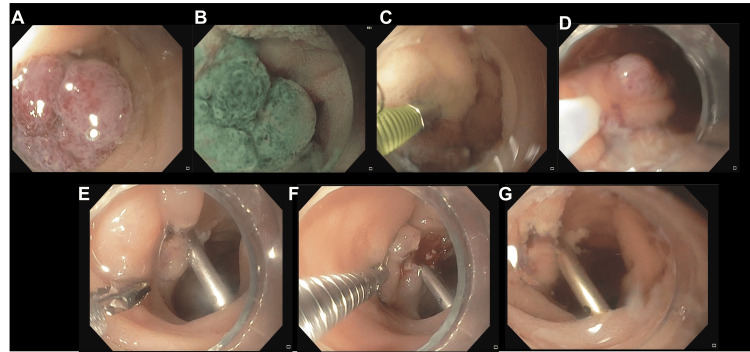
Double-balloon enteroscopy-guided endoscopic management of a small-bowel polypoid venous malformation in blue rubber bleb nevus syndrome. (A) Polypoid lesion visualized under white-light endoscopy. (B) Enhanced mucosal visualization with chromoendoscopy/image-enhanced endoscopy. (C) Submucosal injection to achieve mucosal lift and facilitate safe resection. (D) Snare resection of the lesion. (E) Post-resection defect with endoscopic clip placement for mechanical hemostasis. (F) Post-treatment site showing clips with minimal oozing. (G) Water-immersion view of the post-treatment area confirming clip position and hemostasis.

Based on capsule endoscopy findings, the patient underwent bidirectional double-balloon enteroscopy with endoscopic resection of small-bowel polypoid vascular lesions using a snare technique, followed by clip placement for mechanical hemostasis (Figure 4).

Following these interventions, the patient's clinical status improved markedly. She is currently asymptomatic, with stabilized hemoglobin levels and no further need for transfusions. The constellation of cutaneous lesions, in conjunction with iron-deficiency anemia and the presence of multiple GI vascular malformations, led to a clinical diagnosis of BRBNS.

## Discussion

The management of BRBNS has evolved significantly over the decades. Early reports from the late 20th century [6,7]documented initial approaches to treatment, often involving extensive surgical interventions and limited endoscopic capabilities. In contrast, recent advances in endoscopic techniques have allowed for more precise and less invasive management of the vascular lesions associated with this syndrome, as demonstrated in our case, where endoscopic resection and clip placement were successfully employed.

Several articles emphasize the interplay between persistent iron-deficiency anemia and GI involvement in BRBNS, often managed with endoscopic interventions and other medical therapies [3,8-10]. Additional perspectives on diagnostic and therapeutic complexities, including snare resection, cyanoacrylate, sclerotherapy, and conservative management, have also been described [11-14].

In diagnosing BRBNS, upper and lower endoscopies emerge as the primary modalities due to their ability to directly visualize and manage GI lesions. These procedures are pivotal for diagnosis and immediate therapeutic interventions, such as polypectomy or cauterization of bleeding lesions. However, our review indicates that enteroscopy and video capsule endoscopy become indispensable in many cases, especially when symptoms persist or when lesions are suspected beyond the reach of standard endoscopes [9,11,14,15]. Video capsule endoscopy enables a comprehensive evaluation of the small intestine and helps localize suspected bleeding sources, while device-assisted enteroscopy allows targeted therapy when lesions are identified [3,12].

In our case, using endoscopic clips proved an effective treatment strategy to manage bleeding lesions associated with BRBNS. This approach aligns with the therapeutic strategies reported in several cases, where endoscopic interventions have successfully minimized the bleeding and stabilized hemoglobin levels. Applying hot snare techniques followed by clip placement provided a dual benefit of removing the lesion while securing hemostasis. Figure 4 illustrates the stepwise endoscopic approach (image enhancement, submucosal lift, snare resection, and clip-based hemostasis) used to control bleeding lesions in the small bowel. Mechanical hemostasis was achieved with through-the-scope endoscopic clips (Resolution™ clip; Boston Scientific, Boston, MA, USA), which are particularly advantageous in managing vascular anomalies like the one described by Campos-Murguía and Zamora-Nava [11]. Comparatively, our method mirrors the increasing preference for minimally invasive techniques that offer prompt relief and reduce the need for more extensive surgical procedures, reflecting a less invasive intervention trend [3,15].

The recurrence of GI bleeding and the need for periodic interventions highlighted in various case reports emphasize the necessity for ongoing surveillance and long-term management plans for patients with BRBNS [9,10,16]. This underscores the chronic and potentially debilitating nature of the disease, necessitating a tailored and proactive approach to care. Similarly, our patient demonstrates a favorable prognosis; she is hemodynamically stable and undergoing follow-up. This stability is encouraging and indicates that with continuous monitoring and appropriate interventions, patients can manage symptoms effectively and maintain a good quality of life.

Several publications present alternative therapeutic options for managing BRBNS, including medical treatments for adjunctive or second-line use [17,18]. Another article explores a single-balloon enteroscopy-assisted laparoscopy with endoscopic mucosal resection (EMR), offering a more invasive yet precise approach for lesions not reachable via conventional endoscopy, removing significant lesions inaccessible through traditional endoscopy [19].

This report is limited by the incomplete availability of longitudinal laboratory trends, including hemoglobin and iron studies. In addition, long-term outcomes could not be fully characterized due to loss to follow-up and limited access to outside records. Despite these constraints, the clinical course and endoscopic findings underscore BRBNS as an important consideration in refractory iron-deficiency anemia and obscure GI bleeding.

Taken together, these reports support a pragmatic, stepwise approach to evaluation and follow-up in suspected BRBNS. In patients with unexplained iron-deficiency anemia or obscure GI bleeding, particularly when characteristic cutaneous venous malformations are present, initial assessment should include a complete skin examination and baseline laboratory evaluation (complete blood count and iron studies), followed by bidirectional endoscopy (EGD and colonoscopy). If anemia or bleeding persists despite non-diagnostic upper and lower endoscopy, video capsule endoscopy is appropriate to evaluate the small bowel, with device-assisted enteroscopy (e.g., double-balloon enteroscopy) for localization and endoscopic therapy when feasible. Management is ideally coordinated through a multidisciplinary team (gastroenterology, hematology, dermatology, and surgery/interventional radiology as needed), combining supportive therapy (iron replacement ± transfusion) with targeted endoscopic hemostasis. Surveillance should include periodic monitoring of hemoglobin and iron indices, with repeat endoscopic evaluation guided by recurrent anemia, bleeding, or symptoms.

## Conclusions

BRBNS remains a challenging cause of recurrent GI bleeding due to multifocal venous malformations and variable clinical presentation. This case highlights the value of a multidisciplinary approach and advanced endoscopic therapy in achieving hemostasis and improving clinical status. Ongoing surveillance with clinical follow-up and laboratory monitoring is important to detect recurrence and guide timely reintervention.
